# Using the Baidu Search Index to Predict the Incidence of HIV/AIDS in China

**DOI:** 10.1038/s41598-018-27413-1

**Published:** 2018-06-13

**Authors:** Guangye He, Yunsong Chen, Buwei Chen, Hao Wang, Li Shen, Liu Liu, Deji Suolang, Boyang Zhang, Guodong Ju, Liangliang Zhang, Sijia Du, Xiangxue Jiang, Yu Pan, Zuntao Min

**Affiliations:** 1 School of Social and Behavioral Sciences, Nanjing University, Nanjing, 210023 China; 2 The Johns Hopkins University-Nanjing University Center for Chinese and American Studies, Nanjing, 210093 China; 3 The First Affiliated Hospital with Nanjing Medical University, Nanjing, 210029 China; 4 Department of Medicine, Tongji Hospital, Tongji University, Shanghai, 200065 China; 5 Department of Cardiothoracic Surgery, Shanghai Children’s Hospital, Shanghai Jiao Tong University, Shanghai, 200062 China

## Abstract

Based on a panel of 30 provinces and a timeframe from January 2009 to December 2013, we estimate the association between monthly human immunodeficiency virus/acquired immune deficiency syndrome (HIV/AIDS) incidence and the relevant Internet search query volumes in Baidu, the most widely used search engine among the Chinese. The pooled mean group (PMG) model show that the Baidu search index (*BSI*) positively predicts the increase in HIV/AIDS incidence, with a 1% increase in BSI associated with a 2.1% increase in HIV/AIDS incidence on average. This study proposes a promising method to estimate and forecast the incidence of HIV/AIDS, a type of infectious disease that is culturally sensitive and highly unevenly distributed in China; the method can be taken as a complement to a traditional HIV/AIDS surveillance system.

## Introduction

Initially detected in Yunnan in the early 1990s, HIV/AIDS in China has now spread into 30 provinces^1,2^. At a national level, the HIV/AIDS epidemic maintains a low-prevalence trend; however, in some key regions, such as Yunnan, Sichuan, Henan, Guangxi, and Xinjiang, HIV incidence is extremely high, representing the majority of HIV diagnoses in China^3–7^. Despite the Chinese government’s enormous efforts toward HIV/AIDS prevention and control, the HIV epidemic continues to grow. By the end of 2014, 1.59 per 100,000 population died of AIDS-related illness. Around the country, an estimated 2.96 per 100,000 people were living with HIV, and 2.05 per 100,000 people were identified as AIDS patients^4^. HIV prevention has become one of the most severe challenges for national health and development.

Many claim the detection of HIV/AIDS suffers from drastic underreporting in China^8^. As the HIV epidemic is concentrated among a key affected population, including intravenous drug users, female sex workers, men who have sex with men, and transgender people, the tremendous stigma attached to the disease constitutes a serious impediment to HIV diagnosis, bringing about a widespread fear of HIV/AIDS. As in numerous other countries across the world, much of the development aid has been bogged down by bureaucracy^9^; the expense of HIV confirmation tests and the relative inaccessibility or unavailability of antiretroviral drug therapies result in a large disparity between official and unofficial estimates, particularly in remote rural areas^10^.

Digital surveillance systems provide an innovative and effective way to facilitate faster detection. Built on Internet search engines, these systems can provide real-time information about the emergence and spread of an infectious disease; they are found to have a good congruence with traditional healthcare surveillance systems, especially for diseases such as influenza, dengue, Ebola, malaria, and breast cancer, despite the relevant limitations^11–15^. HIV/AIDS is by no means easy to detect; accurate forecasting of the incidence of HIV/AIDS has a significant impact on the utilization of resources and the establishment of a preventive foundation for future epidemics. Up till now, the effective method in the prediction of HIV epidemics is almost nil.

Time series analysis has been one of the most commonly practiced methods in epidemiology studies^16^. As with many other infectious diseases, changes in HIV/AIDS incidence are influenced by changing time trends, seasonal fluctuations and random disturbances. HIV/AIDS epidemics are concentrated in certain areas in China. The incidence of HIV/AIDS depends largely on the mode of transmission among individuals. Any temporary or permanent protection and control would bring about statistical challenges for prediction. Time series analysis, which consists of many approaches, can effectively deal with such problems (e.g., uncertain geographic coverage and serial correlation). In this study, taking advantage of monthly *BSI* data^17^, we examine the association of *BSI* and the monthly trend of HIV/AIDS incidence in 30 Chinese provinces between 2009 and 2013. Considering the dynamic and varying transmission rates across provinces, we adopt a pooled mean group (PMG) model in the estimation^18^. Our results show that the PMG estimator can provide accurate and reliable predictions of monthly HIV/AIDS incidence.

## Study Methods

### Data source

#### Epidemiological data

The monthly count data of HIV/AIDS incidence was obtained from the Chinese Center for Disease Control and Prevention (China *CDC*) [http://www.chinacdc.cn/], which covers 31 provinces (including four province-level municipalities and five province-level autonomous regions) in China, using a timeframe spanning January 2009 to December 2013.

#### Baidu search data

As reported by the China Internet Network Information Center, more than 95% of Internet users in China made Baidu their first choice among all the search engines. Baidu, the most popular search engine in China, has provided its users with instant access to a gigantic ranking database of numerous keywords from June 2006 onward. The data is available at various levels, provincial or national, with different time units – daily, weekly, monthly or yearly. For ease of analysis, we collected monthly *BSI* [http://index.baidu.com] for each province covering the same period as the *CDC* data. For certain items, *BSI* is calculated based on the sum of the weight of the search volume by the number of search items. *BSI* reflects the absolute Baidu search volume, but is not equivalent to it. In this study, we selected three widely searched-for items: the monthly BSI for “HIV” and “AIDS” (i.e., “” in Chinese, as well as the two English abbreviations), and summed them up, aiming to examine whether *BSI* could provide an effective prediction of real HIV/AIDS incidence. In this study, we removed Xinjiang from the analytical sample, as the Internet connection was turned off in Xinjiang in 2009 and 2010, bringing the reliability of Baidu search data from here into question. This operation resulted in 60 monthly records for each of the 30 provinces in the sample.

### Models

Dynamic heterogenous panel estimation has attracted rising attention in recent years. The short- and the long-run growth effect are often of primary interest. Researchers often formulate traditional regression models to undertake the analysis, if the underlying time series is stationary and exhibits a long-run relationship. However, this stationary assumption, in most circumstances, is not as commonly applicable as has conventionally thought, resulting in misleading estimations. Cointegration has thus become an overriding requirement for any models with non-stationary time series. When cointegration is met, autoregressive distributed lag (ARDL) models are often applied to jointly estimate the short- and long-run effects of the considered variables, as ARDL models can be reparametrized into error correction models (ECM), in which the short-run dynamics are influenced to any deviation from long-run equilibrium^19,20^.

Recent literature has suggested several approaches in the estimation of dynamic heterogenous panel models. A dynamic fixed-effects (DFE) estimator can provide estimations at one extreme, under which all the provinces are pooled. Both slope coefficients and error variances proved to be identical in the long run; only the intercepts are allowed to vary across provinces. If the homogenous slope coefficients or error variances are violated, the DFE estimator would provide inconsistent results. At the other extreme, a mean group (MG) estimator is employed, which impose no cross-province parameter restrictions and allows the estimated coefficients to vary across provinces. The MG estimator can be estimated on a by-province basis, and it is shown to provide consistent estimates of province-specific mean of the long-run coefficients, when the cross-province dimension is large^21^.

The PMG estimator, on the other hand, reflects an intermediate approach that involves both pooling and averaging, offering a variety of appealing features. As a special case of ARDL models, it can provide a valid estimation of whether variables of interest follow the I(0) or I(1) process. As aforementioned, it can also separate short- and long-run relationships between *BSI* and HIV/AIDS incidence. More often than not, short-run dynamics are less likely to share common features across provinces; common features are often revealed in the long run. This insight can be exploited by the PMG estimator, which imposes homogeneity of slope coefficient in a long-run relationship, while allowing the short-run coefficients, such as the intercepts, the speed of adjustment to the long-run equilibrium values and error variances, to be heterogenous across provinces. This slope homogeneity holds when the long-run equilibrium is expected. If not, an alternative estimator (the MG estimator) should be adopted. When neither slope coefficients nor error variances vary across the provinces in the long run, the DFE estimator should be employed. As stated, these three estimators can be incorporated into an error-correction model using the ARDL (p, q). In this analysis, we take the maximum lag as being 1 (i.e., p = 1; q = 1). The times series of *BSI* and *CDC* records are processed by transforming them into logarithm form. Therefore, the model can be written as follows:

1 $${\rm{\Delta }}\mathrm{log}\,CD{C}_{i}={\varphi }_{i}(\mathrm{log}\,CD{C}_{i,t-1}-{\theta }_{0i}-{\theta }_{1i}\,\mathrm{log}\,BS{I}_{i,t-1})-{\delta }_{1i}{\rm{\Delta }}\mathrm{log}\,BS{I}_{i,t-1}+{\varepsilon }_{it}$$

In Equation (1), $\mathrm{log}\,CD{C}_{it}$ refers to the logarithm of *CDC*-reported HIV/AIDS incidence in province *i* at month *t*, and $\mathrm{log}\,BS{I}_{it}$ denotes the logarithm of the HIV/AIDS *BSI*. To rule out possible 0 incidence and 0 searching, we added a very small number, 0.001, to $CD{C}_{it}$ and $BS{I}_{it}$ before taking the logarithm. By taking the log, we transform the highly skewed distributions to distributions close to normal, which helps meet the assumption of inferential statistics. In the model, ${\delta }_{1i}$ is a short-run coefficient of lagged $\mathrm{log}\,BS{I}_{i,t}$, and ${\boldsymbol{\theta }}$ are the long-run coefficients. ${\varphi }_{i}$ is the coefficient of speed of adjustment to the long-run equilibrium; when ${\varphi }_{i}=0$, there would be no long-run relationship. ${\varepsilon }_{i,t}$ stand for idiosyncratic errors at month $t$ in province *i*, which are independent and identically distributed. All three models are estimated using the STATA14.0 command “xtpmg”^22^.

## Results

### Descriptive analysis

**Figure 1:**
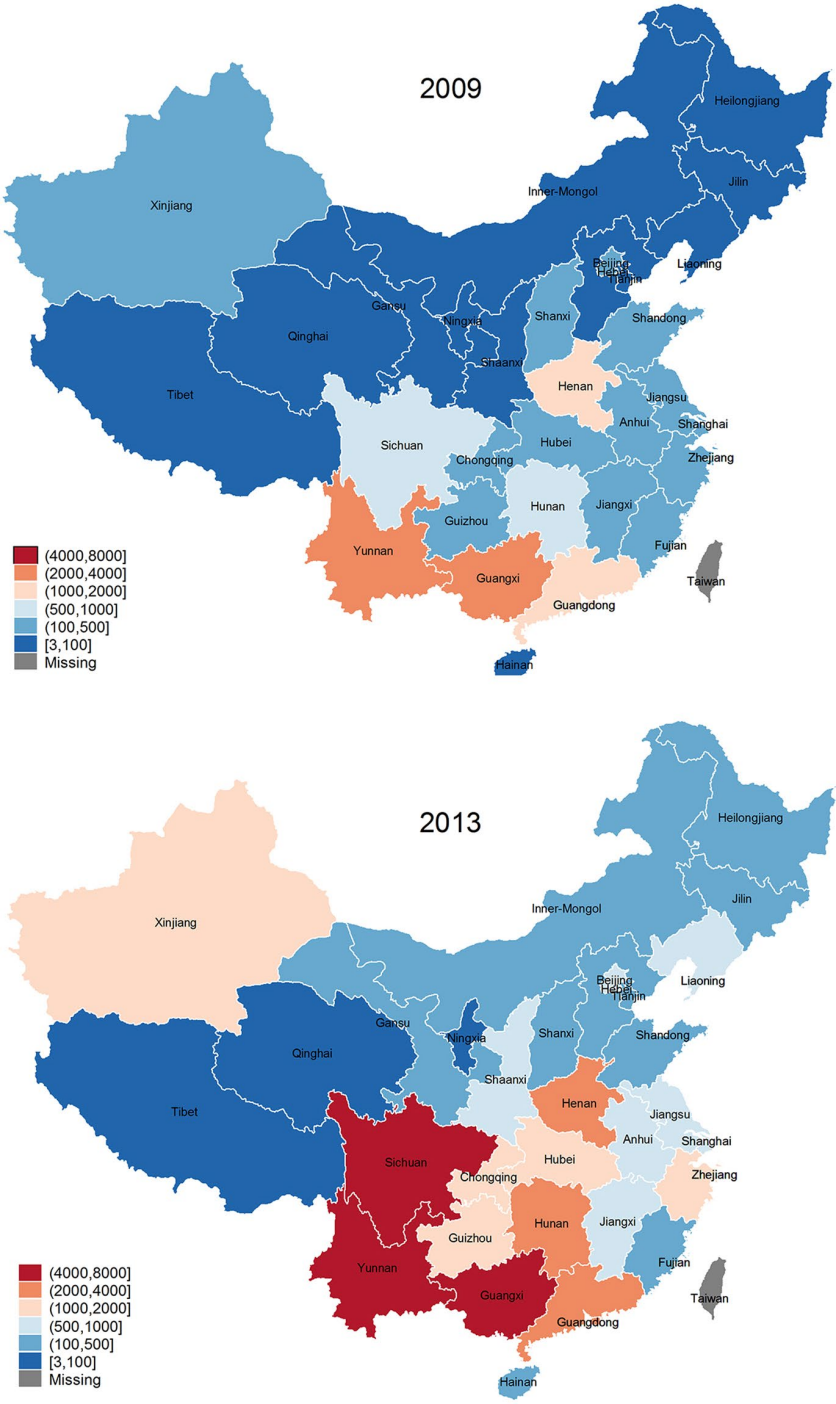
Yearly HIV/AIDS incidence in China by province in 2009 and 2013. The yearly incidence data is calculated by summing up the monthly incidence in 2009 and 2013 from the *CDC*-reported incidence records for each province. To show the trend of provincial HIV/AIDS incidence from 2009 to 2013, we use the “spmap” command in STATA14.0 to draw the map^27^.

According to the monthly reported incidence rates by *CDC*, HIV/AIDS incidence has been growing steadily in China. In 2009, only four provinces had more than 1 per 1,000 population people newly infected with HIV/AIDS; by 2013, the number of provinces had increased to twelve (including Xinjiang). The prevalence of HIV/AIDS is particularly severe in Guangdong, Guangxi, Henan, Sichuan and Yunnan (Fig. 1), which together account for over 60% of HIV/AIDS infection in China.

**Figure 2:**
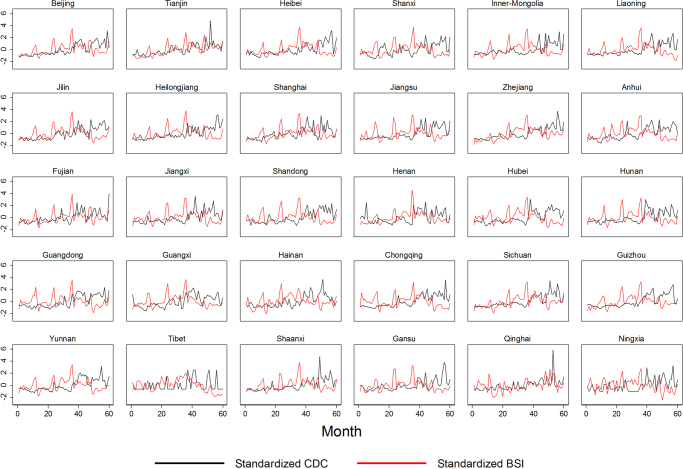
Standardized monthly HIV/AIDS incidence and BSI by province, January 2009–December 2013.

To show the distribution of $BSI$ and $CDC$ record of HIV/AIDS, we present the summary statistics in Table 1. Both $BSI$ and $CDC$ had large variations at national level, where between-province variations accounted for 70.55% and 83.53% of the total variation of the variables of interest respectively (Table 1). To bridge the link between these two variables, we further plotted the provincial monthly trend of $BSI$ and $CDC$ records. To remove the scale effect of the variables, we standardized the $BSI$ and $CDC$ recodes for each province. As Fig. 2 shows, the standardized $BSI$ went hand in hand with the $CDC$ records, indicating the presence of dynamic interactive effects between $BSI$ and the incidence of HIV/AIDS. We also fit naïve bi-variate model for each province, and found that most provinces had moderate correlation coefficients between 0.3 and 0.5 in 2013. Additionally, some seasonal patterns can be revealed in both $BSI$ and $CDC\,$(Fig. 2).

**Table 1:** Descriptive statistics for variables.

Abbr.	Description		Mean	Std. Dev.	Min.	Max.
*CDC*	HIV/AIDS incidence from *CDC*	Overall	73.425	130.066	0.001	970.001
		Between	N/A	110.920	0.601	483.151
		Within	N/A	70.835	−214.725	780.4082
*BSI*	HIV/AIDS Baidu Searching Index	Overall	259.690	131.678	25.001	1027.001
		Between	N/A	122.227	51.334	595.5677
		Within	N/A	53.755	32.124	691.1238

### In-Sample Prediction

The application of the PMG model requires that the data should follow the I(0) or the I(1) process. To test this, we first conducted a stationary test for all the variables in their original form and the first difference. The augmented Dickey-Fuller test shows that, although $\mathrm{log}\,CDC$ and $\mathrm{log}\,BSI$ do not conform to I(0) process in some provinces, both variables are shown to follow I(1) process across all the provinces in China.

To examine the association of monthly $BSI$ and true HIV/AIDS incidence records from January 2009 to December 2013, we estimate Equation (1) with PMG, MG and DFE estimators. As shown in Table 2, the coefficient of error-correction term of the three estimators are all significantly negative, with the magnitude ranging from −0.366 to −0.609 (no smaller than −2), which indicates the existence of a long-run relationship between $BSI$ and $CDC$ (the premise behind the application of the long-run modeling). From DFE to PMG, further to MG, a gradient of decreasing model restrictions is displayed.

**Table 2:** Long- and short-run association of HIV/AIDS *BSI* and HIV/AIDS incidence.

	DFE		PMG		MG	
	Coef.	Std. Error	Coef.	Std. Error	Coef.	Std. Error
Long-run coefficient						
log *BSI*	0.902	0.349	2.089	0.311	2.091	0.381
Short-run coefficient						
Error correction	−0.609	0.022	−0.366	0.033	−0.376	0.034
D. log *BSI*	−0.451	0.222	−0.434	0.168	−0.383	0.229
Constant	−1.213	1.150	−3.221	0.378	−2.721	0.931
Hausman Test			55.80^a^		0.00^b^	
p-value			0.000		0.991	
No. provinces	30		30		30	
No. observations	1800		1800		1800	

(a) Hausman h-test of DFE and PMG estimator; (b) Hausman h-test of PMG and MG estimator.

To choose among these three methods, we conducted the Hausman test. By comparing DFE and PMG, a significant Hausman test suggested that the equalization of the speed of adjustment coefficient and short-run coefficients across provinces was invalid; the PMG estimator was thus preferred. By further comparing the PMG and MG estimators, the insignificant test result revealed that two estimators can produce no different estimation of coefficients; in this regard, long-run slope homogeneity cannot be rejected. Clearly, imposing slope homogeneity somewhat reduced the standard residual of the long-run $\mathrm{log}\,BSI$ coefficient, while the change of the point estimate, which is at the third digit after the decimal point, was minor. Research suggests that only when there is no long-run slopes homogeneity can the MG estimator provide a consistent estimate of the mean of the long-run coefficients. When no long-run slope homogeneity is violated, the PMG estimator is preferred. As in case of slope homogeneity, PMG is consistently proved to offer an efficient and consistent estimate^23^. In this analysis, we therefore chose PMG as the best fitting model. The results show that *BSI* has a positive and significant long-run correlation with CDC-reported incidence (Table 1): a 1% increase of $BSI$ was associated with a 2.089% of increase in HIV/AID incidence. In the short run, the increase of *BSI* index has, to some degree, suppressed the HIV/AID incidence.

**Figure 3:**
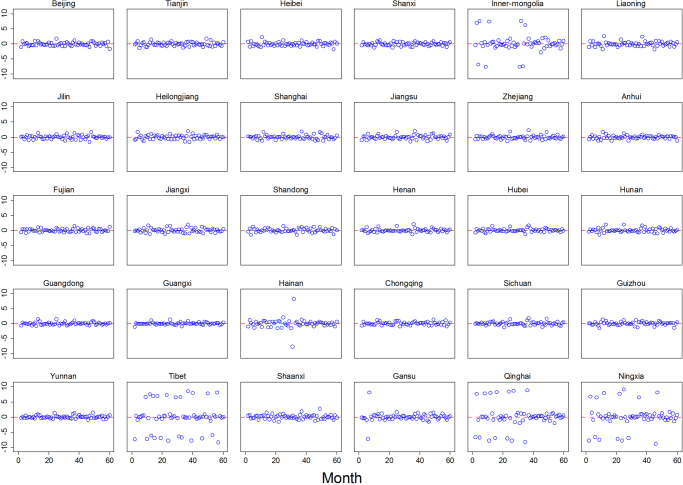
Standard error for the PMG model.

**Figure 4:**
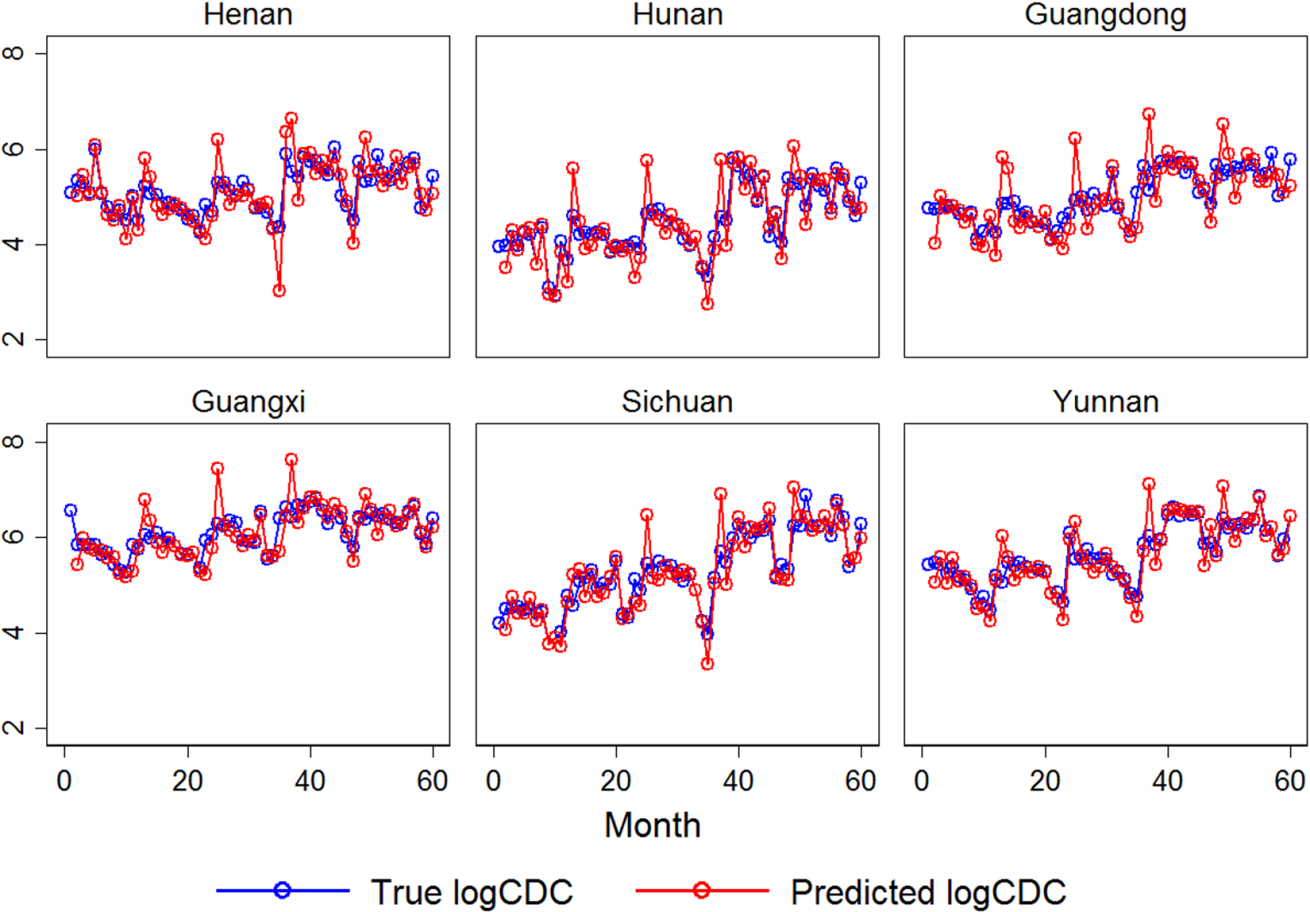
True and predicted logCDC in six provinces with high HIV/AIDS concentration, using the PMG model.

To show visually the goodness-of-fit of PMG estimator, the residual-time plot was drawn (Fig. 3). From Fig. 3, the PMG model fits the HIV/AIDS incidence extremely well. The predicted residuals present no apparent time trend, showing a straight horizontal line at y = 0. For the provinces with more scattered dots, such as Inner-Mongolia, Tibet, Qinghai and Ningxia, the larger errors are primarily due to the zero incidence of HIV/AIDS. When looking into the CDC-reported HIV/AIDS incidence data, these four provinces show the lowest HIV/AIDS incidence levels in China. In epidemiology studies, the aim of prediction is to accurately identify the most at-risk groups and to predict the probability of infections and the speed of their spread in high-risk regions. The government and relevant organizations can therefore react promptly to effectively control the transmission of the disease. To show how well the PMG model can predict HIV/AIDS incidence in the HIV high prevalence area, Fig. 4 was drawn to compare the true and predicted valued of HIV/AIDS incidence (Fig. 4). This figure implies that the HIV/AIDS incidence predicted from the PMG model shows a high congruence of HIV/AIDS incidence reported by CDC.

We also tried various other model specifications. As HIV/AIDS incidence is very low in most of the provinces, the static models (such as the two-way fixed-effects model and the AR(1) error model) have shown to provide satisfactory predictions of HIV/AIDS incidence. All these models indicate a positive and significant association between $BSI$ and $CDC$. Unfortunately, static specification fails to take account of the dynamic features associated with high-incidence provinces; this leads to a large underestimation of HIV/AIDS incidence in these areas. The PMG estimator is, so far, the best estimation we come up with.

### Out-of-Sample Forecasting

To check the performance of the PMG estimator further, we also performed out-of-sample forecasting for HIV/AIDS incidence. For the sake of comparison, we divided the period into two when doing the forecasting. The first period covered monthly data from January 2009 to December 2012 (48 months); the second ranged from January 2013 to December 2013 (12 months). We attempted to use first-period data to estimate the model, and further forecast the HIV/AIDS incidence based on this estimated model for the rest of the months covered in the second period.

In STATA, after running “xtpmg”, we create a new variable with fitted values (“*pr*”) and an error correction term (“*ec*”). The sum of *pr* and *ec* is the predicted log *CDC* of the previous month. Rather than directly forecasting HIV/AIDS incidence, we can thus forecast *pr* and *ec* instead. From Equation (1), log *BSI* as a key predictor of log *CDC* is also pivotal to successful forecasting. As Baidu can provide its users with instant search information, we here assume that *BSI* is given across the whole period. This assumption results in a large simplification of the forecasting process. As long as the *BSI* is known, xtpmg can always give the full range of predicted *pr* values. To obtain the accurate log* CDC* forecast, therefore, the central task becomes how to achieve an accurate forecast of *ec*.

As mentioned, the distribution of HIV/AIDS incidences is highly uneven in China. When employing a dynamic forecast, we adopt a static specification of *ec* in low-incidence areas, and a dynamic specification in high-incidence areas. In terms of the former, we used a province-level fixed-effects model with the control of 1-to-5 lags of *ec* and a linear time trend. We also adjusted for provincial fixed components when forecasting. In terms of the latter, we employed a seasonal autoregressive integrated moving-average (SARIMA) model in each of the high-incidence provinces, where ARIMA(1,0,0)(0,1,0)_12_ was the final model adopted for the dynamic forecasting. This model included one AR term and one seasonal difference of *ec*. The inclusion of the AR term takes care of the first order autocorrelation of *ec*, while the adoption of the seasonal differencing can remove a seasonal random walk type of non-stationarity.

The procedure to forecast $ec$ using fixed-effects models is as follows:

1. Estimate the PMG model using first 48-month observations of each province, and predict $pr$ and $ec$;
2. Employ provincial fixed-effects model to create a forecasting model by regressing predicted $ec$ from step 1 to its 1-to-5 lags and a linear time trend;
3. Predict the fixed component from fixed-effects model and create a provincial average fixed component for forecasting adjustment using the “forecast” command;
4. Store the predicted $pr$ and forecasted $ec$ at month 49; and
5. Create the forecasted log *CDC* at time 49 by summing the predicted $pr$ and forecasted $ec$ at month 49.

We carried out the above five steps recursively until we obtained all the logCDC forecast from month 49 to month 60.

We took a different approach when forecasting $ec$ in provinces with a high incidence of HIV/AIDS using the SARIMA model. The procedure was as follows:

1. Estimate the PMG model using the first 48-month observations of each province, and predict the full range of $pr$ and $ec$ for the first 48 months;
2. Estimate ARIMA (1,0,0)(0,1,0)_12_ model of $ec$ from step 1 using the first 48-month observations, then apply a dynamic forecast for $ec$ of month 49 by using the “arima” command in STATA14.0;
3. Replace the missing value of $ec$ predicted from step 1 with the forecasted $ec$ at month 49; and
4. Create the forecasted log *CDC* at time 49 by summing the $pr$ estimated from step 1 and the $ec$ at month 49.

To obtain all the log *CDC* forecast in the second period, we carried out steps 2–4 recursively.

Notice that when using a fixed-effects model to forecast $ec$, we also update $\,ec$ by running the PMG model recursively. This is because, as a static specification, the fixed-effect model can only fix the province-level time-invariant pattern of $ec$. By doing this, we took account of minor, even nuisance, time-variant changes as much as we could. However, in the ARIMA model, as a dynamic specification, these problems are far less of a concern.

**Figure 5:**
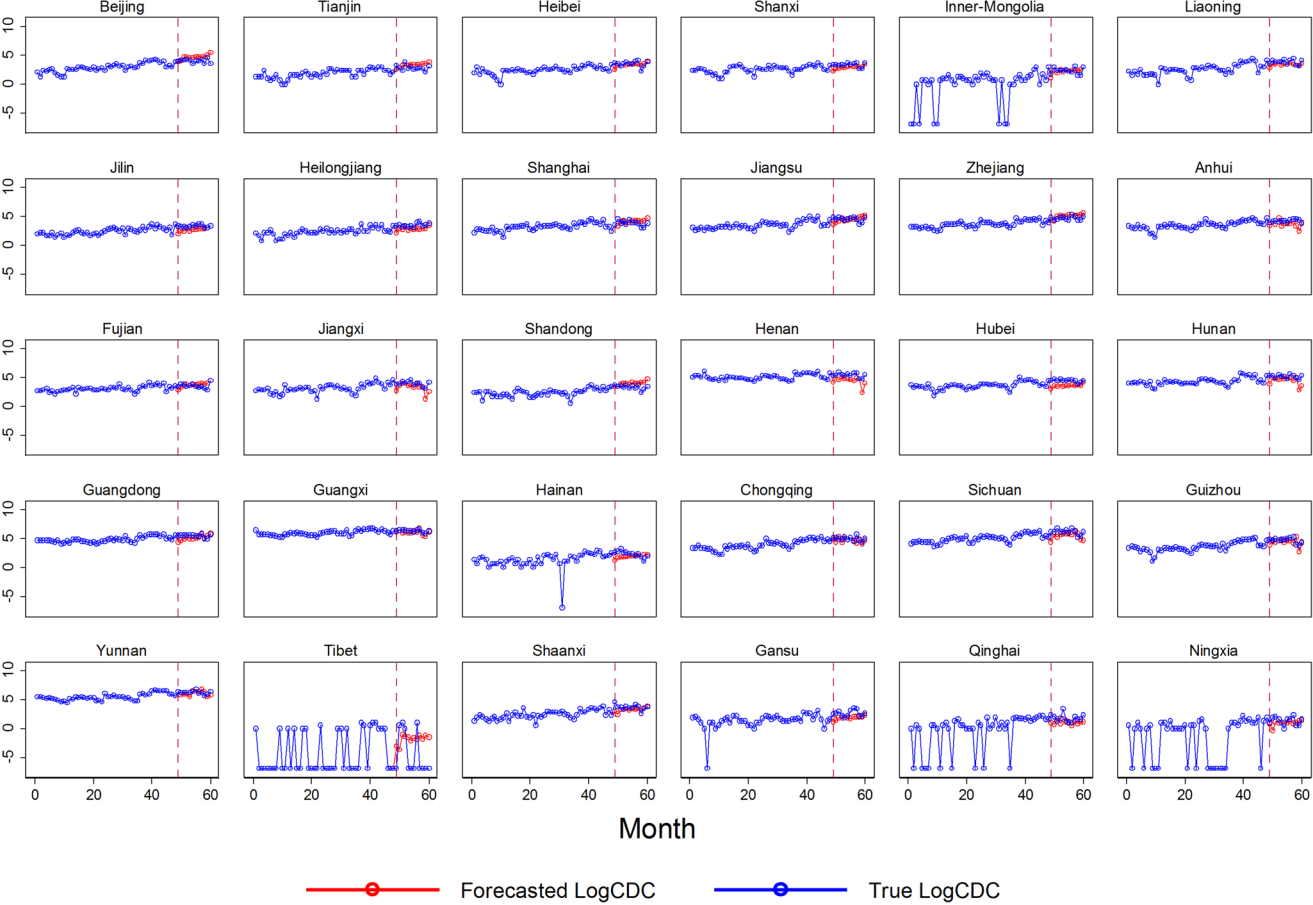
Forecasted log *CDC*. The forecasting is conducted in low and high HIV/AIDS prevalence areas respectively; high prevalence areas include Anhui, Jiangxi, Henan, Hubei, Hunan, Guangdong, Guangxi, Chongqing, Sichuan, Guizhou and Yunnan.

**Figure 6:**
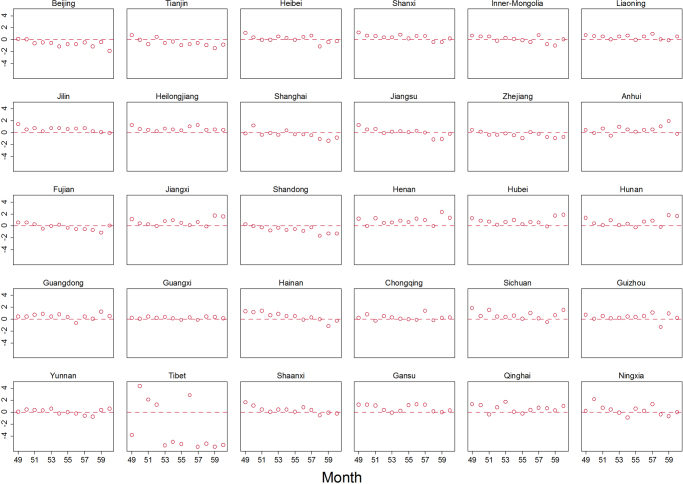
Forecasted error by province.

The forecasting results of HIV/AIDS incidence from January to December 2016 for each province are presented in Fig. 5. Clearly, our strategy, although indirectly, provided satisfactory log *CDC* forecasts, especially for some provinces with high HIV/AIDS incidence (such as Yunnan and Guangxi). However, our forecast contained a relatively large forecast error for Tibet: the province contains several zero incidences over time (Fig. 6). We also tried various other time breakpoints, such as the 30^th^ month (i.e., we forecast the last 30-month records with the first 30-month HIV/AIDS incidence) and the 35^th^ month (i.e., we forecast the last 25-month records with the first 35-month HIV/AIDS incidence); the forecasting results remained consistent.

## Discussion

HIV/AIDS prevalence in China has long been low at the national level, but high in some key regions. This low national prevalence might mask the high prevalence of HIV among specific groups in certain areas. HIV/AIDS can never simply be taken as an infectious disease; it involves a dynamic and interactive correlation between government action and epidemic. More often, it involves ethnic politics in some areas in China. Despite the government’s enormous efforts at HIV prevention, due to the cultural stigma associated with HIV, a deep fear of disclosure undermines the state’s attempts at HIV/AIDS diagnosis, treatment and progression. Globally, it has been widely suggested that the HIV epidemic is underreported. It is therefore necessary to identify some effective and accurate prediction models to assist the detection of HIV/AIDS incidence. Existing studies, however, primarily focus on the identification of HIV transmission type^24^, and attempts to predict HIV/AIDS incidence remain void.

Effective prediction is a prerequisite for prompt HIV prevention. In epidemiology studies, much recent scholarship has drawn attention to the use of Internet search data to assist in the surveillance of some diseases. Taking advantage of Baidu search data, this study attempts to examine whether the *BSI* of HIV/AIDS can be an effective predictor of the detection of province-specific HIV/AIDS incidence in China. The result of the PMG estimation has suggested that the increase of *BSI* in the short run can decrease the HIV/AIDS incidence temporarily, while in the long run, *BSI* has shown a significantly positive correlation with HIV/AIDS incidence.

China experienced a growing epidemic of HIV/AIDS over the time span covered by the analysis. Considering the pervasive variation of HIV transmission type across regions^25^, the rising trend of HIV/AIDS may imply the increasing dominance of one or more types of HIV transmission channels. However, the increase of HIV/AIDS incidence does not necessarily indicate the rapid spread of HIV/AIDS through actual virus transmission. This pattern may imply certain other meanings. One recent research in Hong Kong has suggested that media coverage can predict HIV related search^26^. As scale and intensity of media coverage varies across social contexts, whether media would arouse the similar level search in mainland China remains an open question. Moreover, this trend of HIV/AIDS incidence may reveal the advancement of medical technology, which has improved the accuracy and precision of diagnosis while reducing the cost associated with it. It also reveals the government’s ceaseless efforts at HIV prevention, for example the implementation of relevant health policy and the increase in the health service coverage to areas with high concentration of HIV/AIDS. Regarding the positive long-run association between $BSI$ and $CDC$, the growing awareness of individuals about HIV/AIDS infection is implied. Given the availability of relevant services, individuals actively participating in HIV/AIDS check-ups give impetus to the growing identification of HIV/AIDS. These factors, taken together, contribute to the rising trend of HIV/AIDS incidence as observed. Before taking prompt action, an attempt to predict HIV/AIDS accurately and reliably is necessary.

Our study nevertheless has several limitations that deserve further discussion. First, to achieve the consistent estimation of short-run effects, the application of the PMG model requires no serial correlation of predicted errors. However, not all provinces can satisfy the zero serial correlation assumption made in this analysis. The good thing is that the long-run relationship is of great interest in our study. As Pesaran suggests, given that independent variable x has a finite-order autoregression, it is reasonable to allow for the possible independence between x and error when estimating the long-run coefficient^19^. This being said, despite the presence of serial correlation in some provinces, the long-run coefficient remains consistent and efficient. Second, our prediction contains relatively large predicted errors in the areas with very low HIV/AIDS incidence. It is important to note that HIV/AIDS incidence is closely associated with the accessibility or availability of relevant health services; possible underestimation should be taken into account.

HIV prevention is never easy. As a culturally sensitive disease, HIV/AIDS control and prevention should be conducted in a more strategic way. Internet search data can provide an innovative and cost-effective way to help early detection of potential HIV/AIDS infection, and it can be used as a complement to a traditional HIV/AIDS surveillance system.
